# Anshen Buxin Liuwei Pill, a Mongolian Medicinal Formula, Could Protect H_2_O_2_-Induced H9c2 Myocardial Cell Injury by Suppressing Apoptosis, Calcium Channel Activation, and Oxidative Stress

**DOI:** 10.1155/2022/5023654

**Published:** 2022-01-13

**Authors:** Hai-Ying Tong, Yue Dong, Xian-Ju Huang, Ghulam Murtaza, Yu-Jia Huang, Muhammad Sarfaraz Iqbal

**Affiliations:** ^1^School of Traditional Chinese Medicine, Beijing University of Chinese Medicine, Beijing, China; ^2^Department of Medicine and Pharmacy, College of Pharmacy, South-Central University for Nationalities, Wuhan, China; ^3^Department of Biosciences, COMSATS University Islamabad, Lahore, Pakistan; ^4^Department of Biotechnology, University of Okara, Okara, Pakistan

## Abstract

**Background:**

Anshen Buxin Liuwei pill (ABLP) is a Mongolian medicinal formula which has a therapeutic effect on the symptoms such as coronary heart disease, angina pectoris, arrhythmia, depression and irritability, palpitation, and short breath. However, its bioactivity against cardiac injury remains unclear.

**Methods:**

The protective effect of ABLP was evaluated using H9c2 cells. Cell viability, intracellular Ca^2+^, reactive oxidative indices, and mitochondrial membrane potential (∆ψ) were assessed, respectively. The mRNA levels of Ca^2+^ channel-related genes (DHPR, RyR2, and SCN5A) and oxidative stress-related genes (Keap1, Nrf2, and HO-1) were measured by RT-PCR.

**Results:**

0.5–50 *μ*g/mL ABLP could significantly decrease H_2_O_2_-induced cell injury by suppressing cell necrosis/apoptosis and excess oxidative stress, ameliorating the collapse of ∆ψ, and reducing intracellular Ca^2+^ concentration. Furthermore, 0.5–50 *μ*g/mL ABLP reversed H_2_O_2_-induced imbalance in the mRNA levels of DHPR, RyR2, SCN5A, Keap1, Nrf2, and HO-1 gene in H9c2 cells, which further illustrate the mechanism.

**Conclusion:**

ABLP provided protective and therapeutic benefits against H_2_O_2_-induced H9c2 cell injury, indicating that this formula can effectively treat coronary disease. In addition, the present study also provides an in-depth understanding of the pharmacological functions of ABLP, which may lead to further successful applications of Mongolian medicine.

## 1. Introduction

Cardiovascular disease (CVD) is a leading cause of death globally, contributing to more than 17 million deaths in 2017, of which mortality of coronary heart disease (CHD) is the most prevalent. In China, CHD is the main threat to human death. The morbidity of CHD in China was relatively lower than in Western countries [1]. However, due to the vast population base, about 23 million CHD cases were reported in China in 2016 [1]. Advances in cardiovascular research have identified oxidative stress as an important pathophysiological pathway in the development and progression of heart failure [2].

As an important part of traditional Chinese medicine (TCM), the traditional Mongolian medicine (TMM) system is also being practiced like other ethnic or traditional minority medicines in China on the basis of philosophies and practices of Mongolian culture. The origin of TMM belongs to the early 13^th^ century. Later on, it evolved into a typical medicine system in Inner Mongolia and its neighboring areas [3]. TMM has accumulated a wealth of clinical experiences in the development process based on its unique theoretical systems.

Anshen Buxin Liuwei pill (ABLP), a Mongolian medicinal formula, consisted of 6 medicinal materials including *Bos taurus domesticus* Gmelin (named “Niu-Xin” in Chinese), *Myristica fragrans* Houtt. (named “Rou-Dou-Kou” in Chinese), *Choerospondias axillaris* (Roxb.) Burtt et Hill (named “Guang-Zao” in Chinese), *Eugenia caryophyllata* Thunb. (named “Ding-Xiang” in Chinese), *Aucklandia lappa* Decne. (named “Mu-Xiang” in Chinese), and *Liquidambar formosana* Hance (named “Feng-Xiang-Zhi” in Chinese). TMM medical records consider that ABLP has a therapeutic effect on the symptoms such as coronary heart disease, angina pectoris, arrhythmia, depression and irritability, palpitation, and short breath [4]. Zhang [5] treated 114 patients with angina pectoris with ABLP and compound Danshen tablets, respectively. The results show that compared with compound Danshen tablets, ABLP is better in clinical efficacy and ECG results, and ABLP is more effective in the treatment of angina pectoris. Anlusi [6] randomly divided 112 patients with angina pectoris of coronary heart disease into the treatment group (*n* = 56) and control group (*n* = 56). The control group was treated with metoprolol succinate sustained release tablets, and the treatment group was treated with ABLP for 4 weeks. The results showed that the total effective rate of the treatment group was 94.64%, which was significantly higher than that of the control group (85.71%). The combination of ABLP and metoprolol succinate sustained release tablets can significantly reduce the frequency and duration of angina pectoris, effectively improve the symptoms of patients, and improve the quality of life. However, the pharmacological effect and mechanism need further clarification. Therefore, an ameliorative effect of ABLP on cardiomyocytes injury should be further investigated to improve the development and utilization of the formula. The present study was thus conducted to evaluate the influence of ABLP on H_2_O_2_-induced H9c2 cardiomyocytes injury. The cell apoptosis and viability, intercellular Ca^2+^, and oxidative stress indices, as well as mitochondria function, were detected to discuss the action pathways.

## 2. Materials and Methods

### 2.1. Chemicals and Materials

ABLP were purchased from Ulanhot Zhongmeng Pharmaceutical Co., Ltd. High-glucose Dulbecco's modified Eagle's medium (DMEM) was purchased from Hyclone (Logan, United States). According to the original composition of this formula, 4 fundamental components were detected according to 2020 China Pharmacopoeia by an UltiMate 3000 high-performance liquid chromatography (HPLC) system (Thermo Fisher Scientific Dionex, Sunnyvale, CA, USA). The major compounds in ABLP were detected using the high-performance liquid chromatography (HPLC) method according to the Chinese Pharmacopoeia (2020 edition), and the contents of compounds in ABLP were about 0.34 mg/g dehydrodiisoeugenol, 1.85 mg/g costunolide, 1.88 mg/g dehydrocostus lactone, and 0.64 mg/g gallic acid, respectively.

H_2_O_2_ (lot no.: 20180611) was purchased from Sinopharm Chemical Reagent Co., Ltd. Fetal bovine serum (FBS) was provided by Sijiqing Co., Ltd. (Hangzhou, China). Methylthiazoltetrazolium bromide (MTT) was purchased from Biosharp (Anhui, China). DMSO was purchased from Sinopharm Chemical Reagent Co., Ltd. The lactate dehydrogenase (LDH, lot no.:20201015) activity test kit was purchased from Beijing Solarbio Technology Co., Ltd. The bicinchoninic acid (BCA, lot no.:20438) protein assay kit was bought from Beijing ComWin Biotech Co., Ltd. The malondialdehyde (MDA, lot no.:20201015) contents and superoxide dismutase (SOD, lot no.: 20210225) activity test kit were acquired from Nanjing Jiancheng Bioengineering Institute (Nanjing, China). Reactive oxygen species (ROS, lot no.: 2080620202206) assay kit, mitochondrial membrane potential assay kit with JC-1 (lot no.: 093030301026), apoptosis-Hoechst staining kit 1 (lot no.: 103030301202), and Fluo-4/AM (lot no.:111920201207) were purchased from Beyotime Institute of Biotechnology.

### 2.2. Cell Cultures and Treatment

Rat-derived H9c2 cardiomyocytes were purchased from the Chinese Academy of Sciences Cell Bank, Shanghai. Cells were cultured in DMEM supplemented with 15% FBS at 37°C in 5% CO_2_. Different concentrations of the samples were prepared in serial dilutions, and DMEM was used as the control. H9c2 cells were incubated with 25–200 *μ*M H_2_O_2_ for 0.5 h to choose the appropriate dosage. In addition, the cells were incubated with or without 0.5–50 *μ*g/mL ABLP for 12 h before H_2_O_2_ exposure.

### 2.3. Assessment of Cell Viability

We followed the methods of Ma et al. [7]. Cell survival was observed with a phase-contrast microscope (Olympus, Japan). At the same time, cell viability was evaluated by the MTT method, real-time cell analyzer (RTCA) [8], and LDH contents. The amount of MTT formazan was qualified by determining the absorbance at 490 nm using a microplate reader (Tecan A-5082, Magellan, Austria). As for RTCA technology, background measurements were taken from the wells by adding 50 *μ*l of the same medium to the E-16 plates. RTCA Software Package 1.2 was used to calibrate the plates. Cells were plated at a density of 1 × 10^5^ cells/ml with the fresh medium to a final volume of 200 *μ*l. Cells were incubated for 24 h at 37°C and 5% CO_2_ in the RTCA cradle. When the cell density is sufficient, follow-up experiments are performed. According to the manufacturer's instruction, the level of LDH in cells was detected with a commercially available kit. The absorbance was measured at 450 nm with a microplate reader as described above.

### 2.4. Measurement of MDA Production and SOD Activity

Cells in the logarithmic growth phase were incubated in 6-well plates for 24 h for stabilization; then, the medium was replaced with DMEM with or without 0.5–50 *μ*g/mL ABLP for 12 h. After incubated with 50 *μ*M H_2_O_2_ for 0.5 h, the cells were harvested and sonicated with phosphate buffer saline (PBS) to obtain cell homogenates. The MDA levels and SOD activities were detected at 532 nm and 450 nm by a microplate reader, respectively, according to the manufacturer's instruction. The protein concentrations of lysis buffer were measured by the BCA protein assay kit to standard the concentration of MDA and SOD.

### 2.5. Morphological Assessment of Apoptosis

Hoechst staining kit is a classic and fast and easy method of detecting apoptosis. According to the manufacturer's protocol, H9c2 cells from different treatment groups were washed with PBS and incubated with Hoechst 33258 staining fluid at 37°C for 5 min. The cells' nuclear morphological changes were observed under an Olympus fluorescence microscope (Tokyo, Japan) at 461 nm. The nucleus of normal cells was normal blue fluorescence, while the nucleus of apoptotic cells was dense stained or fragmentary dense stained, with some white color.

### 2.6. Measurement of Mitochondrial Membrane Potential (ΔΨ)

The change in the mitochondrial membrane potential (ΔΨ) was determined using the JC-1 kit. H9c2 cells from different treatment groups were washed with DMEM and incubated with JC-1 (1 *μ*M) in DMEM at 37°C for 20 min. After washing with DMEM, the cells were immediately detected by flow cytometry. Green fluorescence was detected by FL1 channel, red fluorescence was detected by FL2 channel, and the relative proportion of red and green fluorescence was commonly used to measure the proportion of mitochondrial depolarization.

### 2.7. Detection of Intracellular ROS

DCFH-DA fluorescent probe was employed to monitor intracellular ROS. Cells in the logarithmic growth phase were incubated in 6-well plates for 24 h for stabilization; then, the medium was replaced with DMEM with or without 0.5–50 *μ*g/mL ABLP for 12 h and then incubated with 50 *μ*M H_2_O_2_ for 0.5 h. After treatment, the cells were washed with fresh DMEM three times and then incubated with 10 *μ*M DCFH-DA at 37°C for 30 min. Ultimately, the fluorescence intensity of cells was observed under a fluorescence microscope, and data were evaluated using Image J software (National Institutes of Health, Bethesda, MD, USA) [9, 10].

### 2.8. Measurement of Intracellular Calcium Ion ([Ca^2+^]) Levels

Fluo-4/AM was employed as Ca^2+^ indicators to measure intracellular Ca^2+^ concentrations. After treatment, the cells were washed with fresh DMEM three times, and the H9c2 cells were loaded with 5 *μ*mol/L Fluo-4/AM for 30 min at 37°C in the dark. The fluorescence intensity was analyzed by flow cytometry.

### 2.9. RNA Extraction and Real-Time Polymerase Chain Reaction (RT-PCR)

RT-PCR was employed to determine the gene expression. After treatment, the total RNA was extracted from H9c2 cells with the TRIzol reagent (Qiagen, Valencia, CA United States). For mRNA quantification, cDNA was synthesized from 1 *μ*g of total RNA using the PrimeScript^TM^ RT reagent kit (TaKaRa, Dalian, China) following the manufacturer's instructions. Reactions were performed in a 20 *μ*L volume according to the thermal cycler manufacturer's protocol (Rox) using an ABI 7500 RT-PCR system (Applied Biosystems) under the following condition: 30 s at 95°C, followed by 40 cycles of 15 s at 95°C, and 1 min at 57°C or 60°C [11]. The primer sequences used for the amplification of target genes are given in Table 1.

### 2.10. Statistical Analysis

Data analysis and calculation of standard deviation were done by SPSS 18.0 (IBM, US). The results were presented as means ± SEM. The data comparison was performed using a one-way analysis of variance (ANOVA) followed by Dunnett's test to detect intergroup differences. A probability (*P*) value of less than 0.05 was considered statistically significant.

## 3. Results

### 3.1. ABLP Efficiently Potent H9c2 Cells against H_2_O_2_-Induced Cytotoxicity

**Figure 1:**
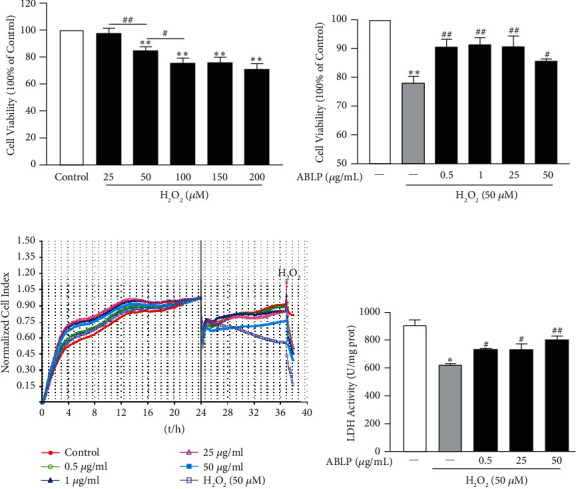
The influence of ABLP on H_2_O_2_-induced cytotoxicity in H9c2 cells. (a) The cell viability influenced by H_2_O_2_ for 0.5 h. (b) MTT analysis of proliferation of H9c2 cells. (c) RTCA technology analysis of proliferation of H9c2 cells using E-16 plates. (d) Effect of ABLP on the LDH level in H9c2 cells subjected to H_2_O_2_. *n* = 6 (*n* the number of experiments). ^*∗*^*P* < 0.05 and ^*∗∗*^*P* < 0.01, the H_2_O_2_ group versus the normal control group. ^#^*P* < 0.05 and ^##^*P* < 0.01, the ABLP group versus the H_2_O_2_ group or H_2_O_2_ group (one dose) versus H_2_O_2_ group (another dose).

As shown in Figure 1(a), the cell viability was dose-dependently reduced in H9c2 cells after incubation with H_2_O_2_ (50–200 *μ*M) for 0.5 h. 50 *μ*M was thus selected as the optimal concentrations of H_2_O_2_ to discuss the influence of ABLP further. MTT assay and RTCA showed that 0.5–50 *μ*g/mL ABLP could significantly increase H_2_O_2_-induced cell injury (Figures 1(b) and 1(c)). LDH is an important marker of cell injury. As shown in Figure 1(d), the content of LDH in cells decreased markedly in the H_2_O_2_ group compared with the control group (*P* < 0.05), indicating apparent release of LDH due to cell damage and split. However, the cell decrease was significantly blocked by preincubation of ABLP in a dose-dependent manner (*P* < 0.05 vs. the H_2_O_2_ group). Together, these findings indicated that ABLP could promote cell survival and reduce cell damage in H9c2 cells subjected to H_2_O_2._

### 3.2. ABLP Strongly Reduced H_2_O_2_-Induced Oxidative Stress and Intercellular ROS Release

**Figure 2:**
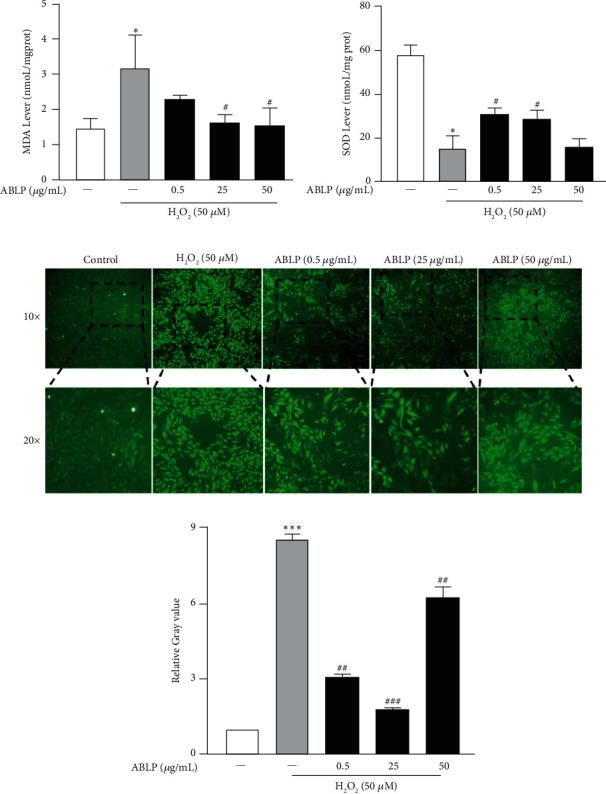
The influence of ABLP on H_2_O_2_-induced oxidative stress and intercellular ROS production in H9c2 cells. (a) Effects of ABLP on MDA content under H_2_O_2_ treatment in H9c2 cells. (b) Effects of ABLP pretreatment on SOD product in H9c2. (c) Fluorescence analysis of intercellular ROS. (d) Relative gray value of ROS. *n* = 3 (*n* the number of experiments). ^*∗*^*P* < 0.05 and ^*∗∗*^*P* < 0.01, the H_2_O_2_ group versus the normal control group. ^#^*P* < 0.05 and ^##^*P* < 0.01, the ABLP group versus the H_2_O_2_ group.

The oxidative stress and antioxidant activity markers detected in the study are shown in Figure 2. MDA contents of the H_2_O_2_ group were significantly elevated in Figure 2(a) (*P* < 0.05 vs. the control group). Conversely, SOD activity was markedly declined in Figure 2(b) (*P* < 0.01 vs. the control group). However, 25 *μ*g/mL and 50 *μ*g/mL ABLP pretreatment significantly reduced MDA elevation and enhanced SOD activity (*P* < 0.05 vs. the H_2_O_2_ group). At the same time, an increase in intercellular ROS occurred in the H_2_O_2_ group with a stronger DCF fluorescence signal compared with the control group, which could be attenuated by ABLP treatment (Figures 2(c) and 2(d)).

### 3.3. ABLP Prevented H_2_O_2_-Induced Apoptosis and Mitochondria Damage

**Figure 3:**
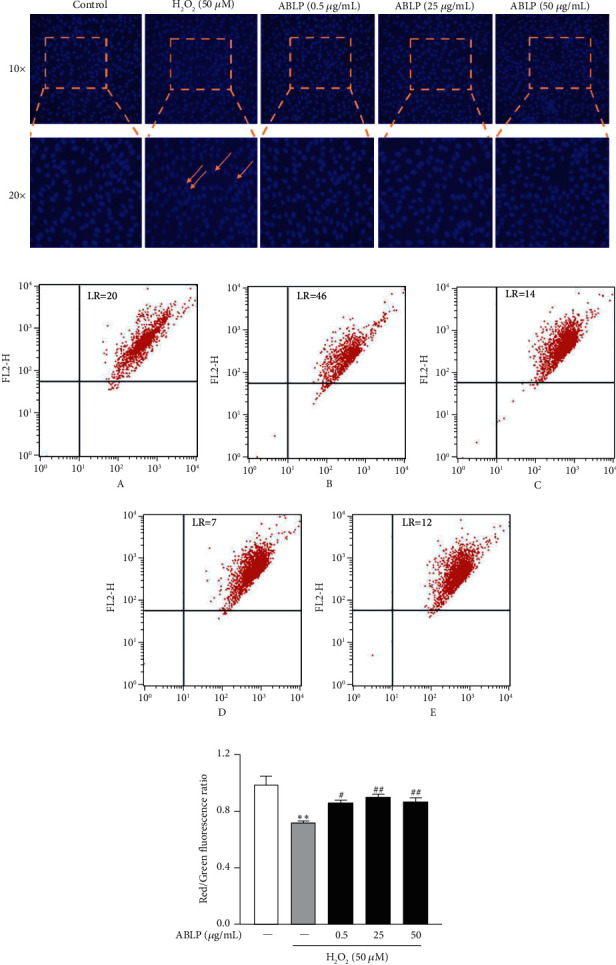
The effect of ABLP on H_2_O_2_-induced apoptosis and mitochondrial membrane potential changes. (a) Apoptosis detected by staining with Hoechst 33258. (b) Flow cytometry analysis of H_2_O_2_-induced mitochondrial injury. A, control; B, H_2_O_2_ (50 *μ*M); C, ABLP (0.5 *μ*g/mL); D, ABLP (25 *μ*g/mL); E, ABLP (50 *μ*g/mL). (c) The red-green fluorescence ratio analysis of mitochondrial depolarization. *n* = 3 (*n* the number of experiments). ^*∗*^*P* < 0.05 and ^*∗∗*^*P* < 0.01, the H_2_O_2_ group versus the normal control group. ^#^*P* < 0.05 and ^##^*P* < 0.01, the ABLP group versus the H_2_O_2_ group.

As shown in Figure 3(a), H9c2 cells with Hoechst staining showed uniform blue fluorescence in the control group under fluorescence microscopy. In contrast, H_2_O_2_-induced apoptotic cells showed hyperchromatic and dense fluorescent particles within the massive apoptotic nuclear cytoplasm. Thus, pretreatment of ABLP (0.5–50 *μ*g/mL) could attenuate H_2_O_2_-induced apoptosis. On the other hand, JC-1 staining showed that ΔΨ drastically decreased in the H_2_O_2_ group than in the control group, which could be significantly blunted by ABLP pretreatment (Figure 3(b)).

### 3.4. ABLP Partially Blunted the Intracellular Ca^2+^ Induced by H_2_O_2_

**Figure 4:**
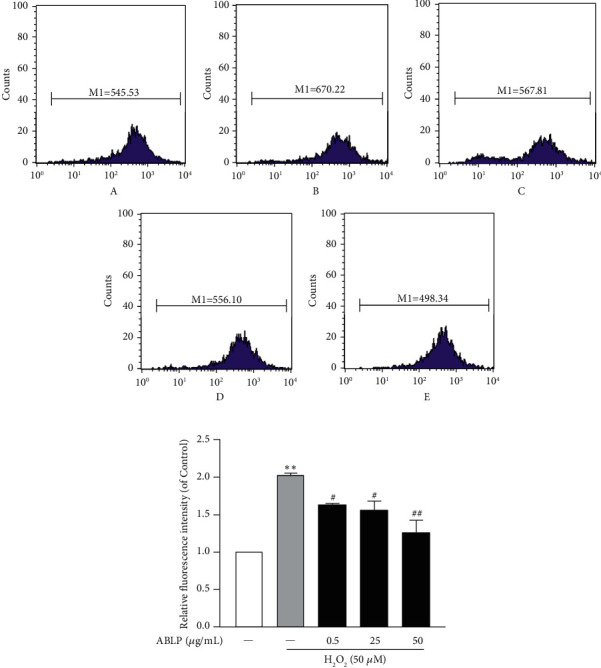
The influence of ABLP on the intracellular Ca^2+^ increase by H_2_O_2_. (a) Flow cytometry analysis of H_2_O_2_-induced intracellular Ca^2+^. A, control; B, H_2_O_2_ (50 *μ*M); C, ABLP (0.5 *μ*g/mL); D, ABLP (25 *μ*g/mL); E, ABLP (50 *μ*g/mL). (b) Analysis of relative fluorescence intensity. *n* = 3 (*n* the number of experiments). ^*∗*^*P* < 0.05 and ^*∗∗*^*P* < 0.01, the H_2_O_2_ group versus the normal control group. ^#^*P* < 0.05 and ^##^*P* < 0.01, the ABLP group versus the H_2_O_2_ group.

As shown in Figure 4, the Ca^2+^ concentration was significantly enhanced in the H_2_O_2_ groups than the control group (*P* < 0.01), which could be decreased by pretreatment with ABLP (0.5–50 *μ*g/mL).

### 3.5. Effect of ABLP on the Gene Levels Related to Ca^2+^ Channel and Oxidative Stress Pathway

**Figure 5:**
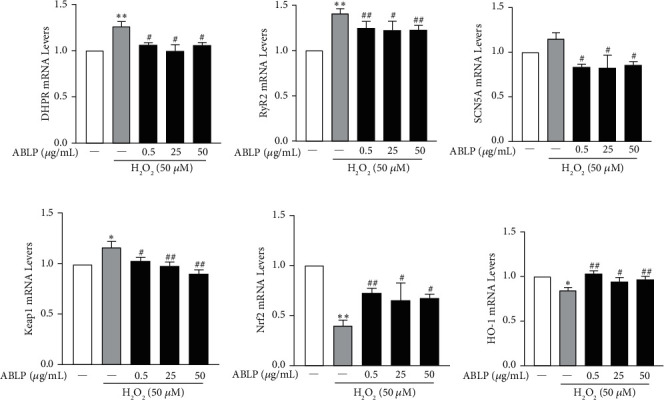
Effect of ABLP pretreatment on related gene mRNA expression in H9c2 cells. (a–c) The mRNA expression of Ca^2+^ channel; (d–f) The mRNA expression of the Keap1-Nrf2/HO-1 pathway, *n* = 3 (*n*, the number of experiments). ^*∗*^*P* < 0.05 and ^*∗∗*^*P* < 0.01, the H_2_O_2_ group versus the normal control group. ^#^*P* < 0.05 and^##^*P* < 0.01, the ABLP group versus the H_2_O_2_ group.

As shown in Figure 5, the mRNA expression levels of DHPR, RyR2, and SCN5A in the H_2_O_2_ group increased compared with the control group, suggesting that H_2_O_2_ could upregulate L-type voltage-dependent Ca^2+^ channels. Conversely, compared with the H_2_O_2_ group, 0.5–50 *μ*g/mL ABLP pretreatment significantly downregulated the mRNA expressions of DHPR, RyR2, and SCN5A (*P* < 0.05).

The influence of ABLP on the oxidative stress pathway is shown in Figure 5. Compared with the control group, incubation of H_2_O_2_ could upregulate the mRNA level of Keap1 and decrease the mRNA levels of Nrf2 and HO-1. Compared with the H_2_O_2_ group, 0.5–50 *μ*g/mL ABLP pretreatment could significantly reversely regulate the mRNA levels of the three genes (*P* < 0.05).

## 4. Discussion

The damage of cardiomyocyte results from the combined action of multiple factors, among which oxidative stress is one of the leading and direct drivers of cell damage. During oxidative stress, the imbalance between the free radicals and ROS can be observed and lead to harmful effects on the body. Reactive oxygen plays an integral role in both myocardial injury and repair. All biological molecules are destroyed by the solid oxidative activity of ROS, especially cells damaged by lipid peroxidation [12]. Calcium is one of the most crucial intracellular messengers; it functions to translate extracellular stimuli into intracellular signaling pathways that ultimately regulate cellular development, survival, differentiation, and gene expression [13]. Thus, any factors or stimuli that disrupt this well-defined Ca^2+^ balance give rise to heart disease, including arrhythmia [14].

It is well-known that intracellular calcium overload could enhance intracellular ROS production. The augmented ROS directly attacks SERCA2 to decrease its activity, increasing the open probability of RyRs [15, 16] to give rise to [Ca^2+^] elevation in damaged cells [17] and then causing arrhythmias by apoptosis [18–20].

**Figure 6:**
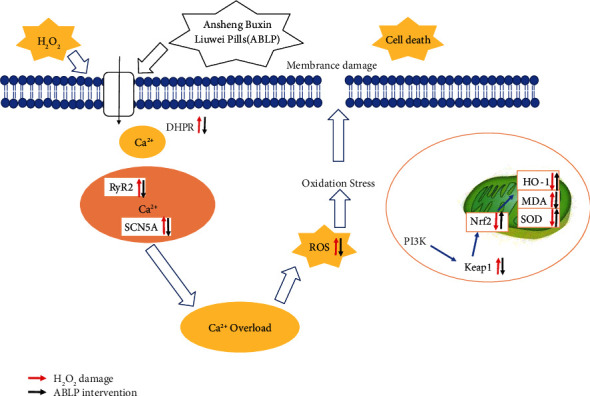
The cardiac protective mechanism of ABLP on H_2_O_2_-exposed H9c2 cells.

Although previous research showed the cardioprotective effects of ABLP in a clinic, the essential mechanism of the myocardial protection of ABLP has not yet been fully explained. The present study revealed that 0.5–50 *μ*g/mL of ABLP could protect H9c2 cells against H_2_O_2_-induced H9c2 cells injury via markedly promoting cell viability, inhibiting cell apoptosis, downregulating excessive oxidative indices, preventing calcium overload, and restoring mitochondrion functions. The protective mechanism of ABLP is shown in Figure 6. Intracellular calcium and ROS overload can lead to myocardial cells damage. Many studies have indicated that sarcoplasmic reticulum is the main calcium pool of myocardial cells, and there are two calcium release channels, RyR channel and IP3R channel, in the sarcoplasmic reticulum. Since RyR density is significantly higher than IP3R, RyR plays a key role in regulating the cardiac arousal systolic conjugate [7]. As the important targets of the calcium channel, DHPR, RyR2, and SCN5A regulate the concentration of Ca^2+^ together and maintain body balance. Our results showed that H_2_O_2_-induced H9c2 oxidative damage could lead to the upregulation of the three genes, resulting in Ca^2+^ overload. Conversely, pretreatment of ABLP could decrease the Ca^2+^ concentration via downregulation of DHPR, RyR2, and SCN5A levels. The endogenous antioxidant pathway regulates the cellular redox homeostasis, i.e., the Nrf2/Keap1/ARE pathway. Nrf2 presented in the cytoplasm is restricted to enter into the nucleus during normal biological conditions through its binding to Keap1.

In contrast, during induced conditions, Nrf2 gets dissociated from Keap1 and enters the nucleus to activate a range of antioxidant genes and enzymes [21]. Therefore, under the status of oxidative stress, Keap1 mRNA will be ascending and Nrf2 and HO-1 mRNA descending. Our study confirmed the protective effect of ABLP against myocardial injury via the Nrf2/Keap1/ARE pathway. However, myocardial injury can deteriorate to various cardiovascular diseases, and it has a relatively unique mechanism of action in some cardiac diseases, which also needs further research and exploration.

## 5. Conclusion

Taken together, ABLP provided protective and therapeutic benefits against H_2_O_2_-induced H9c2 cell injury, suggesting that this formula could be effective for the treatment of coronary disease. In addition, the present study provided a deeper understanding of the pharmacological functions of ABLP, which may lead to further successful applications of Mongolian medicine.

## Figures and Tables

**Table 1:** Primer sequences used for the amplification of target genes.

Gene	Forward	Reverse	Annealing temperature
*β*-Actin	ACCGTGAAAAGATGACCCAG	TCTCAGCTGTGGTGGTGAAG	57°C or 60°C
Nrf2	AACCTCCCTGTTGATGACTTC	CGACTTTATTCTTACCTCTCCT	57°C
Keap1	GCTATGATGGCCACACTTTTCT	GTTGTCAGTGCTCAGGTATTCC	57°C
HO-1	ACGCATATACCCGCTACCTG	CCAGAGTGTTCATTCGAGCA	57°C
DHPR	CATCTTTGGATCCTTTTTCGTTCT	TCCTCGAGCTTTGGCTTTCTC	60°C
RyR2	TGCTGCGAGCCGGG	TGGCGGTGGCGTAGGA	60°C
SCN5A	CACCCTCAACCTCTTCATCG	CTTCTTCTGCTCCTCCGTCA	60°C

## Data Availability

The data used to support the findings of this study are included within the article.

## Supplementary Materials

Supplementary Materials Graphical abstract.
