# Supplementary material for: Anshen Buxin Liuwei Pill, a Mongolian Medicinal Formula, Could Protect H2O2-Induced H9c2 Myocardial Cell Injury by Suppressing Apoptosis, Calcium Channel Activation, and Oxidative Stress
Source: Evid Based Complement Alternat Med. 2022 Jan 13;2022:5023654. doi: 10.1155/2022/5023654 (PMC8843775; doi:10.1155/2022/5023654)
Supplement: Supplementary Materials — Graphical abstract. [file 5023654.f1.pdf]

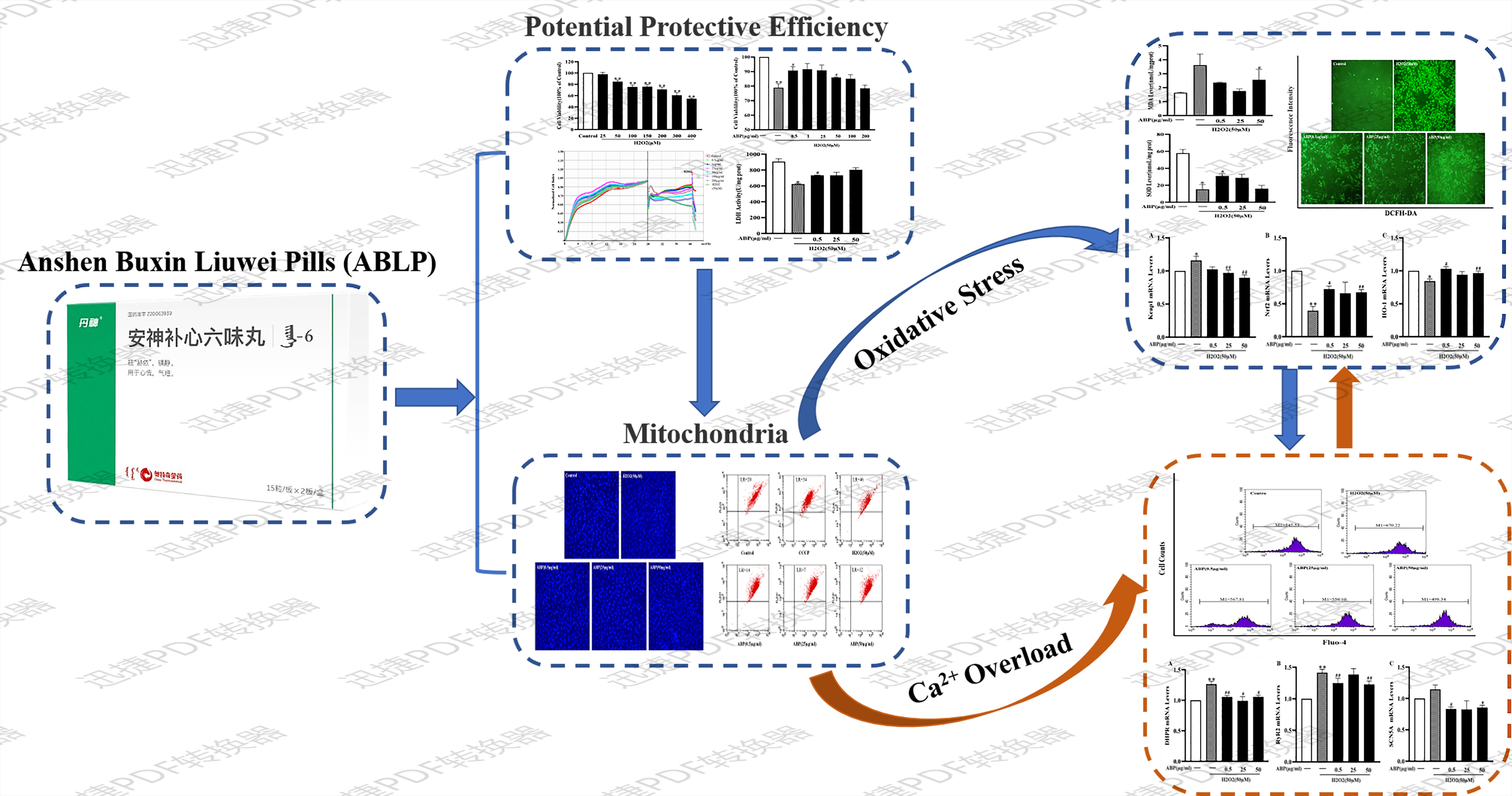
Anshen Buxin Liuwei Pills (ABLP)
安神补心六味丸
Potential Protective Efficiency
Mitochondria
Oxidative Stress
Ca2+ Overload
DCFH-DA
Fluo-4
